# Molecular Characterization of a Novel Intracellular ADP-Ribosyl Cyclase

**DOI:** 10.1371/journal.pone.0000797

**Published:** 2007-08-29

**Authors:** Dev Churamani, Michael J. Boulware, Timothy J. Geach, Andrew C.R. Martin, Gary W. Moy, Yi-Hsien Su, Victor D. Vacquier, Jonathan S. Marchant, Leslie Dale, Sandip Patel

**Affiliations:** 1 Department of Physiology, University College London, London, United Kingdom; 2 Department of Pharmacology, University of Minnesota Medical School, Minneapolis, Minnesota, United States of America; 3 Department of Anatomy and Developmental Biology, University College London, London, United Kingdom; 4 Department of Biochemistry and Molecular Biology, University College London, London, United Kingdom; 5 Center for Marine Biotechnology and Biomedicine, Scripps Institution of Oceanography, University of California at San Diego, La Jolla, California, United States of America; Research Institute for Children and the Louisiana State University Health Sciences Center, United States of America

## Abstract

**Background:**

ADP-ribosyl cyclases are remarkable enzymes capable of catalyzing multiple reactions including the synthesis of the novel and potent intracellular calcium mobilizing messengers, cyclic ADP-ribose and NAADP. Not all ADP-ribosyl cyclases however have been characterized at the molecular level. Moreover, those that have are located predominately at the outer cell surface and thus away from their cytosolic substrates.

**Methodology/Principal Findings:**

Here we report the molecular cloning of a novel expanded family of ADP-ribosyl cyclases from the sea urchin, an extensively used model organism for the study of inositol trisphosphate-independent calcium mobilization. We provide evidence that one of the isoforms (SpARC1) is a soluble protein that is targeted exclusively to the endoplasmic reticulum lumen when heterologously expressed. Catalytic activity of the recombinant protein was readily demonstrable in crude cell homogenates, even under conditions where luminal continuity was maintained.

**Conclusions/Significance:**

Our data reveal a new intracellular location for ADP-ribosyl cyclases and suggest that production of calcium mobilizing messengers may be compartmentalized.

## Introduction

Changes in cytosolic Ca^2+^ are indispensable for normal cell function [1]. This is not surprising given the multiple molecular targets for this ion. In many cells, Ca^2+^ elevations result from the mobilization of intracellular Ca^2+^ stores by Ca^2+^ mobilizing messengers produced in response to extracellular cues [1]. Inositol trisphosphate is the best characterized of these molecules [1]. In addition, cyclic ADP-ribose and nicotinic acid adenine dinucleotide phosphate (NAADP), which were first discovered in the sea urchin egg [2], also play prominent roles in generating complex Ca^2+^ signals [3], [4]. Cyclic ADP-ribose regulates ryanodine receptors on the (sarco)endoplasmic reticulum to mediate Ca^2+^-induced Ca^2+^ release [5], whereas NAADP probably targets novel Ca^2+^ channels [6] located on newly described acidic Ca^2+^ stores [7], although this notion is somewhat controversial [8]. Quite remarkably, both of these messenger molecules are synthesized *in vitro* by the same family of enzymes – the ADP-ribosyl cyclases [9]. These proteins are thus likely to play key roles in Ca^2+^–dependent function.

ADP-ribosyl cyclases have been cloned from *Aplysia* [10], mammals [11], [12] and more recently *Schistosoma* [13]. Little is known concerning the cellular distribution of the *Aplysia* enzyme whereas the mammalian homologues, CD38 (a type 2 membrane protein) and CD157 (a GPI-anchored protein) are both cell surface antigens with their catalytic sites exposed to the extracellular space [14]. The *Schistosoma* enzyme too, appears to be a GPI-anchored plasma membrane protein [13]. These locations are perplexing given the cytosolic location of both the substrates for these enzymes and the Ca^2+^ channels targeted by their products. One attractive hypothesis to explain the apparent “topology paradox” is that cyclic ADP-ribose is produced extracellularly in response to secreted NAD, followed by its influx into the cell via nucleoside transporters, including possibly CD38 itself [15]. Alternatively, CD38 may be internalized upon stimulation [16]. Indeed, in addition to its plasma membrane distribution, CD38 has been found in several intracellular locations including the nucleus [17].

Intriguingly, although agonist-evoked cyclic ADP-ribose production is abolished in the pancreas of CD38 knockout mice [18], [19], underscoring the importance of CD38 in generating cyclic ADP-ribose *in vivo*, basal levels appear unchanged in several tissues of these animals [20]. These data provide strong evidence for the existence of CD38-indpendent cyclic ADP-ribose-synthesizing enzymes. Indeed, novel membrane-associated ADP-ribosyl cyclase activities have been characterized in the brain of CD38 deficient mice [21] and other (CD38 replete) cells [22]–[26]. Moreover, early studies using sea urchin eggs, described an unusal cyclic GMP-sensitive ADP-ribosyl cyclase activity located within the cytosol [27]–[29]. Cytosolic ADP-ribosyl cyclase activity has also been characterized in several mammalian tissues [25], [30]–[33], including the brain [34]. In all of the above cases however, molecular correlates are lacking. Thus, our molecular understanding of ADP-ribosyl cyclases is far from complete. This is particularly so in the sea urchin where many fundamental aspects of the physiology of both cyclic ADP-ribose and NAADP have been defined in gametes [35], [36] and found applicable to a range of other cells across several phyla.

Here we report the first molecular cloning of a family of sea urchin ADP-ribosyl cyclases. Our data show that one of these isoforms is a soluble luminal protein located within the endoplasmic reticulum. We propose compartmentalized production of calcium mobilizing messengers by this novel ADP-ribosyl cyclase.

## Results

### Molecular Cloning of a new Family of Sea urchin ADP-ribosyl Cyclases

**Figure 1:**
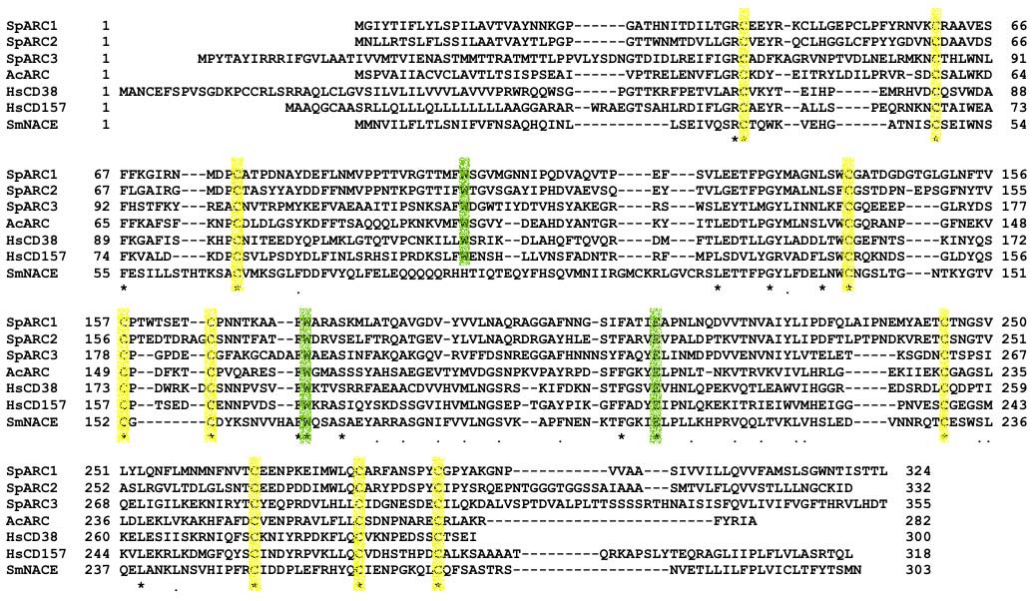
A new family of sea urchin ADP-ribosyl cyclases. A, Sequence alignment of the predicted coding regions of three ADP-ribosyl cyclases isolated from *Stronglycentrotus purpuratus* cDNA libraries (termed SpARC1-3) and representative members of the ADP-ribosyl cyclase family. Conserved cysteine and catalytic residues are highlighted in yellow and green, respectively. Abbreviations: Ac, *Aplysia californica*; Hs, *Homo sapiens*; Sm, *Schistosoma mansoni*.

ADP-ribosyl cyclase activity was first described in the sea urchin egg twenty years ago [2] but molecular information regarding these enzymes in this major model system has not been forth-coming. Here we report the molecular cloning of three sea urchin ADP-ribosyl cyclases, we have named SpARC (*Strongylocentrotus purpuratus ADP-ribosyl cyclase*) 1–3. All isoforms were cloned by PCR from ovary and testes cDNA libraries. The putative open reading frame for SpARC1 corresponded to a 324 amino acid peptide with a molecular weight of 35 kDa, whereas SpARC2 and SpARC3 were predicted to be longer peptides with molecular weights of 36 kDa and 40 kDa, respectively. Multiple sequence alignments of SpARCs with each other and other members of the ADP-ribosyl cyclase family revealed modest sequence similarity at the amino acid level (Fig. 1A, Table S1). For example, SpARC1 and SpARC2 are 66% similar whereas their similarity to SpARC3 is lower at 34 and 36%, respectively (Table S1). Indeed, SpARC1 and SpARC2 appear more similar to *Aplysia californica* ADP-ribosyl cyclase and mammalian CD157 (up to 41% similarity) than to SpARC3 (Table S1). Despite the limited amino acid sequence homology, there is absolute conservation of a glutamate and two tryptophan residues shown previously to be crucial for substrate binding and catalytic activity in other ADP-ribosyl cyclases (Fig. 1A, green residues) [37]. Moreover, all 10 cysteine residues found in *Aplysia* ADP-ribosyl cyclase are also conserved in the sea urchin homologues (Fig. 1A, yellow residues).

Hydropathy analysis indicated that all three proteins possessed hydrophobic N and C termini (Fig. S1). The N terminal sequences were analyzed using the SignalP algorithm [38]. For SpARC1 and SpARC2, a signal peptide was predicted whereas for SpARC3 the sequence conformed better to a signal anchor (Table S2). This analysis is consistent with sea urchin ADP-ribosyl cyclases entering the secretory pathway. In accord, several consensus sites for N-glycosylation (N-{P}-[ST]-{P}) are present in all three proteins (Table S2). We also analyzed the C terminal sequences for potential GPI anchors using the big PI predictor [39]. High scores were obtained for both SpARC2 and SpARC3 but not SpARC1 (Table S2). Overall, the architectures of SpARC2 and SpARC3 are similar to CD157 and CD38, respectively whereas SpARC1 appears to represent a novel ADP-ribosyl cyclase. Indeed, the C terminus of SpARC1 is the most hydrophobic of the isoforms (Fig. S1) and consistent with the presence of a possible trans-membrane domain. Because of the potentially unusual nature of SpARC1, subsequent efforts were focussed on this isoform.

### SpARC1 is Glycosylated

**Figure 2:**
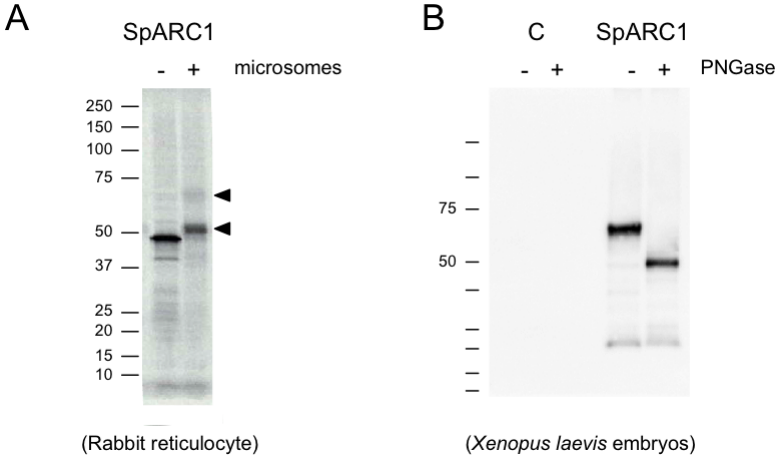
SpARC1 is glycosylated. A, *In vitro* translation. mRNA coding for C terminally *myc*-tagged SpARC1 was translated in the presence of [^35^S]methionine using rabbit reticulocyte lysate. Autoradiogram of translation reactions performed in the absence (−) or presence (+) of canine pancreatic microsomes. B, Expression of SpARC1 in *Xenopus laevis* embryos. Western blot analysis using an anti-*myc* antibody of homogenates prepared from control embryos (C) or embryos injected with mRNA for *myc*-tagged SpARC1. Samples were either mock treated (−) or digested with PNGase F (+) prior to analysis (left). Migration of molecular mass markers (in kDa) is shown on the left of the panels.

For expression studies, we generated a C-terminally *myc*-tagged SpARC1 construct. We first performed *in vitro* transcription/translation studies using the rabbit reticulocyte system. Given the presence of a putative N terminal signal peptide, translation reactions were performed with or without acceptor membranes (Fig. 2A). Autoradiographic analysis of translation reactions resolved by SDS PAGE, using the reticulocyte lysate alone, indicated that *in vitro*-transcribed mRNA drove the expression of a 46 kDa radiolabeled protein (Fig. 2A). The size of the translated product was similar to the predicted size of the tagged construct (47.2 kDa). In the presence of microsomes, slower migrating protein bands were observed (Fig. 2A, arrows). These data are consistent with the presence of a signal peptide driving entry of SpARC1 in to the secretory pathway for its subsequent glycosylation.

We next expressed *myc*-tagged SpARC1 in *Xenopus laevis* because embryos and oocytes from this model system are extensively used for the study of secretory pathway proteins [40]. We first analyzed homogenates prepared from late blastulae that had been injected at the 2 cell stage with mRNA for *myc*-tagged SpARC1. Western blot analysis using an anti-*myc* antibody revealed the expression of a 61 kDa protein (Fig. 2B, right). No signal was obtained in un-injected control embryos (Fig. 2B, left). To determine whether the increased apparent molecular mass of the expressed construct, relative to its predicted size (see above), was due to N-glycosylation, homogenates were treated with PNGase F. Enzyme treatment reduced the molecular mass of SpARC1 to 46 kDa (Fig. 2B, right). These data show that SpARC1 is glycosylated.

### SpARC1 is a Soluble Luminal Protein

**Figure 3:**
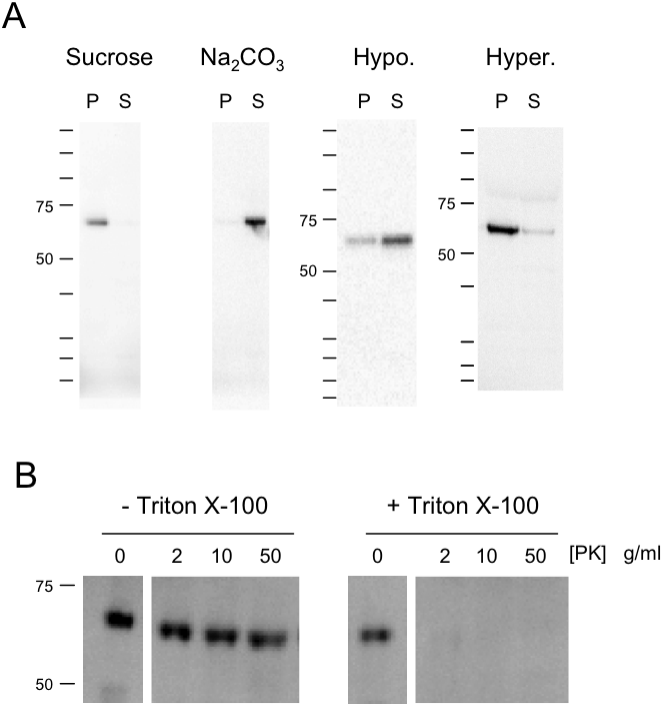
SpARC1 is a soluble luminal protein. A, Sub-cellular fractionation. *Xenopus laevis* embryos expressing *myc*-tagged SpARC1 were homogenized in a sucrose-containing buffer and diluted in to the same buffer (C), a carbonate-containing buffer (Na_2_CO_3_) or a hypertonic buffer (Hyper.). In other experiments the embryos were freeze-thawed and sonicated in a hypotonic buffer (Hypo.). All samples were centrifuged and the resulting pellets and supernatant fractions analysed by Western blotting. B, Proteolytic protection. *Xenopus laevis* embryos expressing *myc*-tagged SpARC1 were homogenized in a sucrose-containing buffer and the homogenates incubated with or without 1% v/v Triton X-100. Samples were then treated with the indicated concentration of proteinase K before Western blot analysis.

To explore the subcellular distribution of SpARC1, we examined pellet and supernatant fractions for immunoreactivity following ultracentrifugation of crude embryo homogenates prepared in the presence of sucrose (Fig. 3). Consistent with the entry of SpARC1 in to the secretory pathway (see above), SpARC1 immunoreactivity was recovered in pellet fractions and not in the supernatant containing the cytosol. Somewhat surprisingly however, treatment of homogenates with Na_2_CO_3_, which strips peripheral proteins and lyses vesicles resulted in near complete solubilisation of SpARC1 (Fig. 3A). Thus, SpARC1 is unlikely to be an integral membrane protein and is either peripherally attached to the membrane or a soluble luminal protein. To distinguish these possibilities, we took 3 approaches. First, embryos expressing SpARC1 were freeze-thawed and sonicated in hypotonic medium. Following this treatment, a substantial fraction of immunoreactivity translocated to the supernatant fraction (Fig. 3A). These data suggest that SpARC1 is a soluble luminal protein. Second we examined the effect of high salt on the distribution of the enzyme. As shown in Fig. 3A, SpARC1 was largely resistant to the hypertonic solution and recovered predominantly in the pellet fraction after treatment (Fig. 3A). This result again supports a luminal location for SpARC1. Finally, we examined the susceptibility of SpARC1 to proteolytic attack. Incubation of homogenates with Proteinase K resulted in the disappearance of SpARC1 immunoreactivity but only when the homogenates were pre-treated with detergent (Fig. 3B). These data provide further evidence that SpARC1 is a soluble vesicular protein.

### SpARC1 is not Secreted

**Figure 4:**
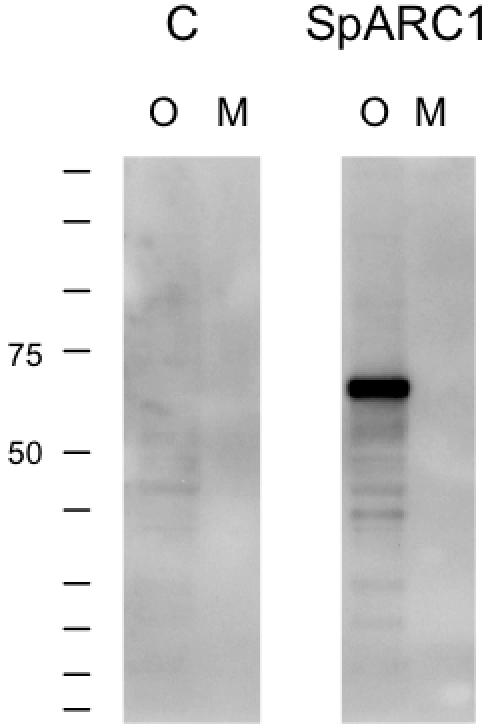
SpARC1 is not secreted. Control *Xenopus laevis* oocytes or oocytes injected with *myc*-tagged SpARC1 were cultured overnight and Western blots for both the oocytes (O) and culture media (M) performed. 0.5 oocyte equivalents were loaded in each lane.

Given that SpARC1 enters the secretory pathway (due to its glycosylation) and is soluble (based on the above fractionation studies), we next examined whether the protein was secreted. For these experiments, we expressed SpARC1 in *Xenopus laevis* oocytes and examined the medium in which the oocytes were cultured for immunoreactivity (Fig. 4). Intriguingly, analysis of equivalent volumes of oocyte homogenates and media did not reveal the presence of SpARC1 in the latter. These data clearly indicate that SpARC1 is not a secreted protein. The molecular weight of SpARC1 (65 kDa) expressed within the oocyte was similar to that observed in embryos (Figs. 3) consistent with its glycosylation.

### SpARC1 Localises to the Endoplasmic Reticulum

**Figure 5:**
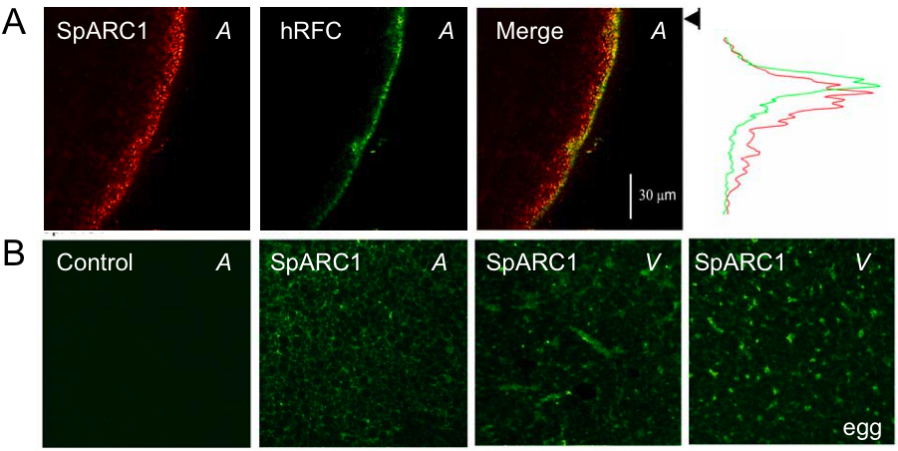
SpARC1 localizes to the endoplasmic reticulum in *Xenopus laevis* oocytes and eggs. A, Confocal fluorescence images of *Xenopus laevis* oocytes co-expressing *myc*-tagged SpARC1 (red) and GFP-tagged human reduced folate carrier (hRFC, green). SpARC1 expression was detected by immunocytochemistry using an anti-myc primary antibody and an FITC labeled secondary antibody whereas hRFC was detected by GFP fluorescence. A plot of the individual emission intensities with depth averaged along a 5 µm wide section (arrowed line) in the overlay image is shown on the right. B, Lateral (‘xy’) images of SpARC1 expression (green) in oocytes and in eggs (right). The control image was obtained from an identically processed un-injected oocyte. Images were captured from the animal (*A*) and vegetal (*V*) poles as indicated.

To determine the sub-cellular distribution of SpARC1 in more detail, we localised the expressed protein in oocytes and eggs by immunocytochemistry (Fig. 5). In these experiments, nuclear plasmid injections were performed to minimize mis-localisation of the mRNA. Co-expression of the human reduced folate carrier tagged with GFP [41], which is a plasma membrane protein, together with SpARC1, led to cell surface expression of the former as expected (Fig. 5A). However, SpARC1 immunoreactivity was broader and distributed deeper into the cell than the tagged carrier, with peak intensity occurring beneath the oolemma within the oocyte cortex (Fig. 5A). Higher resolution images of the animal pole revealed a discrete, reticular pattern of staining in the expressing oocytes, reminiscent of the cortical endoplasmic reticulum (Fig. 5B). The observed distribution was specific since little staining was observed in control un-injected oocytes that were processed identically (Fig. 5B). In the vegetal pole, SpARC1 was also expressed in the endoplasmic reticulum, most clearly evidenced by staining in annulate lamellae, elongated structures which are endoplasmic reticulum sub-domains [42] (Fig. 5B). Because the endoplasmic reticulum is known to undergo marked re-organisation during oocyte maturation [42], we also examined the distribution of SpARC1 in eggs. Consistent with its targeting to the endoplasmic reticulum, SpARC1 was found in discrete patches in the vegetal hemisphere of eggs, which are known to be formed from the endoplasmic reticulum during progesterone-induced maturation [42]. These data suggest that in stark contrast to other known ADP-ribosyl cyclases, SpARC1 is an endoplasmic reticulum enzyme.

**Figure 6:**
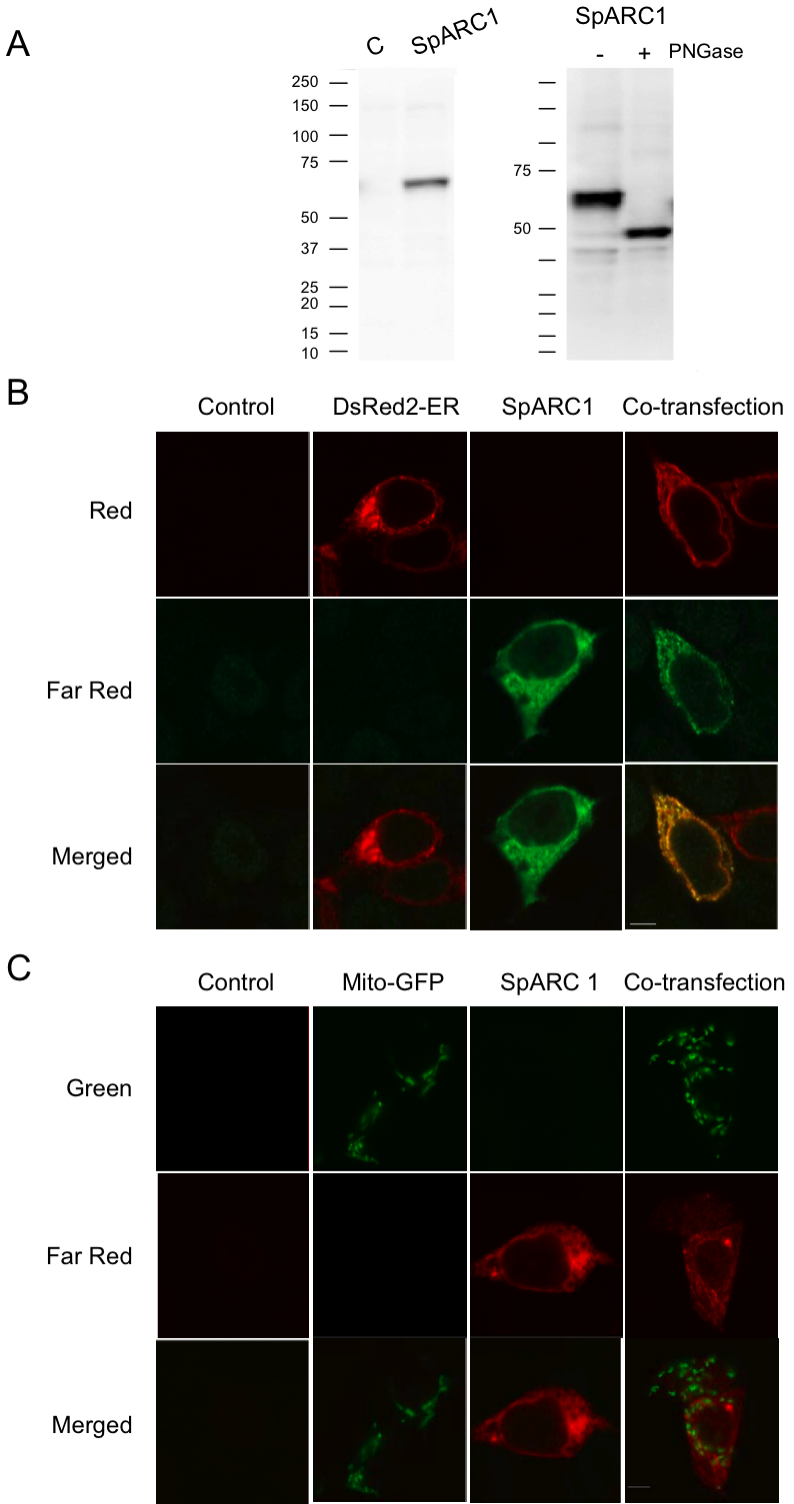
SpARC1 localizes to the endoplasmic reticulum in HEK cells. A, Western blot analysis using an anti-*myc* antibody of homogenates prepared from mock transfected cells (C) or cells transfected with *myc*-tagged SpARC1 (left). PNGase F treatment (right) was performed as described in Fig. 2. B–C, Expression of *myc*-tagged SpARC1 and DsRed2-ER (B) or mitochondrial GFP (C) in HEK cells transfected with the indicated plasmid or plasmid combination. SpARC1 expression was detected by immunocytochemistry using an anti-myc primary antibody and a Cy5-labelled secondary antibody, whereas the endoplasmic reticulum and mitochondria were visualized by fluorescence of the DsRed2 and GFP reporters, respectively. Results from control, mock-transfected cultures are also shown. Images were captured at the wavelengths corresponding to the colours marked at the side of the figure.

SpARC1 localisation was further characterised in an independent expression system. Western blot analysis of human embryonic kidney (HEK) cells transfected with SpARC1 identified a 61 kDa protein the mobility of which was PNGase F-sensitive (Fig. 6A). As in the oocytes and eggs, SpARC1 was localised to an intracellular reticular structure (Fig. 6B). There was no appreciable staining in mock-transfected cells. To identify the organelles to which SpARC1 was targeted, we co-transfected SpARC1 with fluorescent marker proteins for the endoplasmic reticulum and mitochondria. Immunocytochemical analysis revealed clear colocalisation of SpARC1 with DsRed2-ER (Fig. 6B), but not mitochondrial GFP (Fig. 6C). These data provide further evidence that SpARC1 is a novel ADP-ribosyl cyclase targeted to the endoplasmic reticulum.

### SpARC1 is Catalytically Active

**Figure 7:**
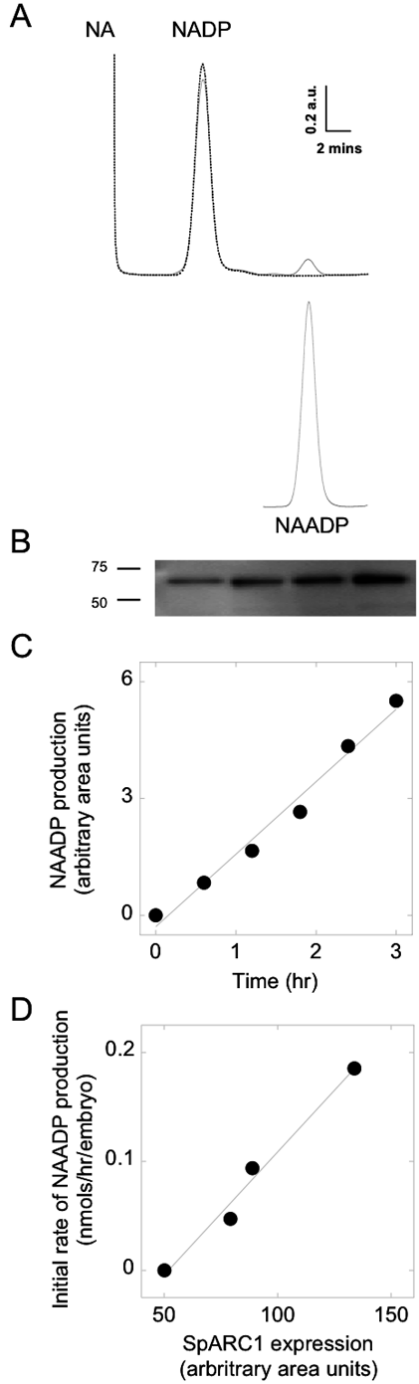
SpARC1 catalyses NAADP production. A, Hypotonic lysates from *Xenopus laevis* embryos were incubated with NADP (1 mM) and nicotinic acid (NA, 50 mM) at pH 4 for 21 h and the resulting mixtures separated by HPLC. The top chromatograms are from lysates prepared from control embryos (dashed lines) and embryos injected with *myc*-tagged SpARC1 mRNA (solid line). The elution of authentic NAADP is shown below. B, Western blot comparing SpARC1 expression in 4 independent embryo preparations. C, Production of NAADP following incubation of a typical embryo homogenate with substrates for the indicated times. D, A plot of the initial rate of NAADP production (measured over 3 h) against the level of SpARC1 expression (determined by densitometric analysis of Western blot data for the preparations analysed in B).

To determine whether SpARC1 is catalytically active, hypotonic lysates prepared from *Xenopus laevis* embryos expressing SpARC1 were examined for base-exchange and cyclization activities. Incubation of the homogenates with a maximal concentration of NADP and nicotinic acid at acidic pH resulted in the synthesis of a product that co-eluted with authentic NAADP (Fig. 7A, solid trace). These data show that SpARC1 is capable of the base-exchange reaction. No endogenous activity could be detected from control un-injected embryos (Fig. 7A, dashed trace). Although the expression of SpARC1 varied considerably between embryo batches (Fig. 7B), the initial rate of NAADP production, which was linear over the first three hours (Fig. 7C), correlated well with the amount of protein detectable on Western blots (Fig. 7D).

**Figure 8:**
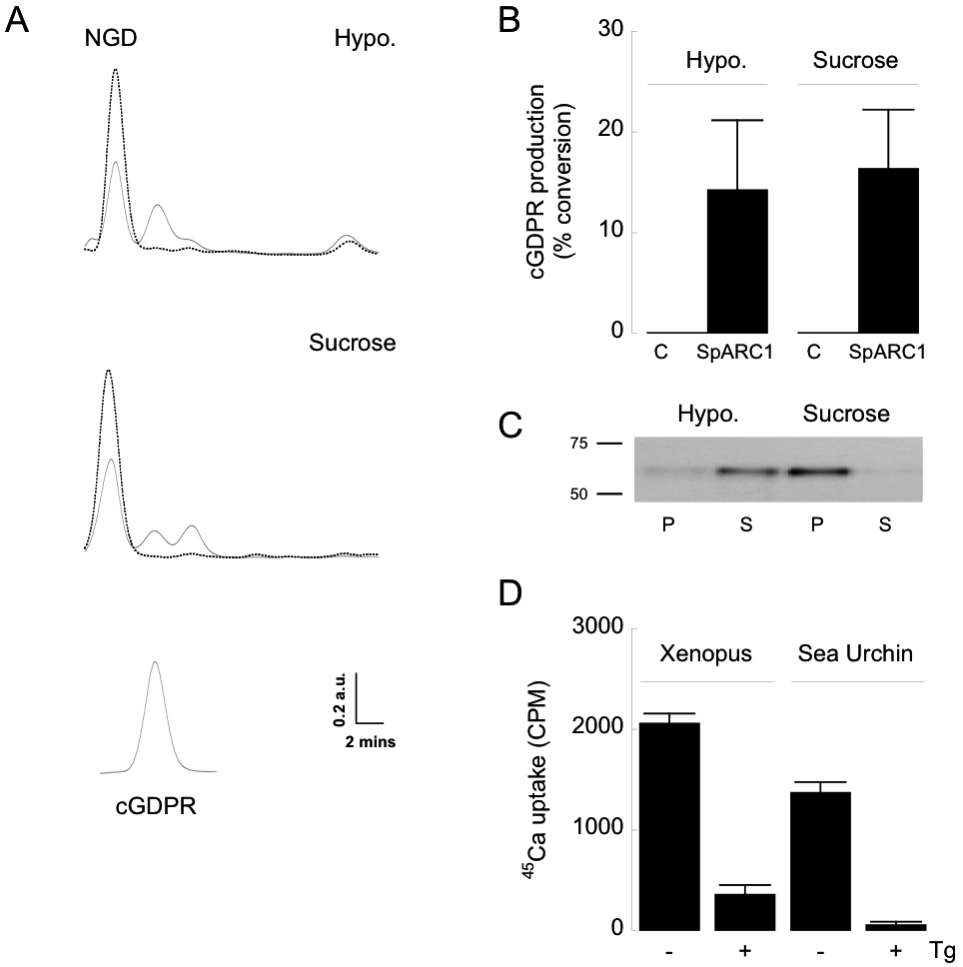
SpARC1 catalyses cyclic GDP-ribose production. Homogenates from *Xenopus laevis* embryos were incubated with 1 mM NGD for 21 h at pH 7.2 and the resulting mixtures separated by HPLC. A, Representative chromatograms from lysates prepared from control embryos (dashed lines) and embryos injected with *myc*-tagged SpARC1 mRNA (solid line). Both hypotonic lysates (Hypo.; top) or homogenates prepared in sucrose-containing medium (Sucrose; middle) were used. The elution of authentic cyclic GDP-ribose is shown below. B, Pooled data (mean+/−standard error of the mean) from 3 experiments comparing the amount of cyclic GDP-ribose produced using either control (C) or SpARC1-expressing preparations. C, Western blot analysis of *myc*-tagged SpARC1 expression in pellet (P) and supernatant (S) fractions prepared following centrifugation of the two reaction mixes from a typical experiment described in A at the end of the incubation period. D, ^45^Ca flux uptake by homogenates prepared from *Xenopus* embryos (in sucrose-containing media) or sea urchin eggs either in the absence (−) or presence (+) of the sarco-endoplasmic reticulum calcium ATPase inhibitor, thapsigargin (Tg; 1 µM). Data are presented as means±standard deviation of triplicate samples from one of 3 independent experiments.

SpARC1-dependent cyclization was also readily demonstrable in this heterologous expression system. In these experiments, we used the NAD analogue NGD which when cyclized forms a metabolically resistant product that is easier to detect than cyclic ADP-ribose [43]. Incubation of embryo homogenates expressing SpARC1 with NGD for 21 h, resulted in the synthesis of a product that coeluted with cyclic-GDP-ribose (Fig. 8A, top). Modest production was detectable over the first 3 hours (data not shown) but more readily resolved after more prolonged incubations as shown. Again, no activity was measurable in control homogenates (Fig. 8A). These data provide strong evidence that SpARC1 is a constitutively active, *bonafide* ADP-ribosyl cyclase.

### Luminal SpARC1 can Access its Substrate

Given the normally luminal location of SpARC1, we assayed homogenates for cyclase activity under conditions that preserved vesicle architecture. Thus, activity measurements were repeated using homogenates prepared in the presence of sucrose (see Fig. 8A, bottom). Remarkably, as summarised in Fig. 8B, cyclic GDP-ribose production was readily detectable under these conditions and not different to that observed using hypotonic lysates prepared from the same embryo batch. These data indicate that SpARC1 can readily access its substrates despite its compartmentalisation.

To assess the continuity of the endoplasmic reticulum in our homogenates, we first examined the distribution of SpARC1 in the activity assay mixes at the end of the incubations. As shown, SpARC1 immunoreactivity was confined to the pellet and not supernatant fractions upon centrifugation (Fig. 8C) providing evidence that cyclase activity detected was not a result of the enzyme leaking from the vesicles during the experiments. To probe the integrity of the endoplasmic reticulum vesicles to smaller solutes, we incubated the homogenates with ^45^Ca in the presence of an ATP regeneration system. Active uptake of the radiolabel was readily demonstrable and inhibited by the SERCA inhibitor, thapsigargin (Fig. 8D). Results from three independent experiments indicated that 70±13% of the uptake was in to the endoplasmic reticulum. Similar results were obtained using sea urchin egg homogenates in which 95±3% (n = 3) of the ^45^Ca uptake was thapsigargin-sensitive (Fig. 8D). These data show that endoplasmic reticulum vesicles in the embryo homogenates are sealed and capable of accumulating a small ion. Thus, accessibility of substrates to SpARC1 is unlikely to result from non-specific leakiness of the endoplasmic reticulum to small solutes such as NGD. Furthermore cyclase activity was also detectable following shorter incubations (3h) where any possible vesicle breach would be minimal (data not shown).

## Discussion

The sea urchin has proved an excellent model for the study of inositol trisphosphate-independent Ca^2+^ mobilization [2], [44]–[47]. ADP-ribosyl cyclase activity was first detected in egg homogenates leading to the identification of both cyclic ADP-ribose [2], [44] and NAADP [2], [45]. Additionally, levels of NAADP were first measured in sperm [46] and shown to increase at fertilization [47]. However, despite the undisputed importance of the sea urchin in the study of both cyclic ADP-ribose- and NAADP-mediated Ca^2+^ signaling - findings quickly extended to other systems - no molecular details of ADP-ribosyl cyclases have been available in this major animal model. Here we report for the first time the molecular cloning of three ADP-ribosyl cyclases from *Strongylocentrotus purpuratus*. These findings extend those in mammals where only two ADP-ribosyl cyclases (CD38 and CD157) have been described at the molecular level, although activity measurements suggest others [22]–[32]. Our data then provides the most comprehensive molecular description of ADP–ribosyl cyclases in a single organism to date.

A major finding of our study is that SpARC1 localizes to the endoplasmic reticulum. This is based on our immunocytochemical analysis of heterologously expressed SpARC1 in eggs and oocytes of *Xenopus laevis* and also HEK cells (Figs. 5–6). This location is entirely consistent with early studies by Lee and colleagues who identified ADP-ribosyl cyclase activity in a “light” microsomal fraction in sea urchin eggs [28]. Retention of SpARC1 within the endoplasmic reticulum is unlikely to be an expression artifact, because similar results were obtained in two disparate cellular expression systems. Although highly expressed proteins can accumulate within the endoplasmic reticulum, this is usually due to misfolding/aggregation. As we have shown, SpARC1 was catalytically active and capable of both base-exchange and cyclization reactions (Figs. 7–8). Indeed, despite modest sequence similarity between the sea urchin proteins and other ADP-ribosyl cyclases, critical residues identified in CD38 and ADP-ribosyl cyclase from *Aplysia* that dictate structure and function are well conserved in all of the sea urchin proteins (Fig. 1). These include cysteine residues and tryptophan 103 (SpARC1 numbering), which notably is not found in the *Schistosoma* enzyme [48].

The location of SpARC1 clearly contrasts with that of CD157 and the *Schistosoma* homologue, which are both GPI-anchored plasma membrane proteins. CD38, like CD157, is also a cell surface antigen although there are reports of CD38 expression in organelles [16], [17], [25] at least in cells of non- hemopoietic origin [49]. Notably, immunoelectron microscopy reveals the presence of CD38 within the endoplasmic reticulum of mammalian neurons [50] consistent with the sub cellular location of SpARC1. Also of note are reports of unidentified intracellular ADP-ribosyl cyclase activities in the sarcoplasmic reticulum of several mammalian muscle types [22]–[24] again consistent with our findings. Taken together, these data raise the possibility that localization of ADP-ribosyl cyclases to the (sarco-) endoplasmic reticulum is unlikely to be restricted to sea urchins.

Membrane bound ADP-ribosyl cyclase activity has previously been described in several cell types including sea urchin egg and sperm homogenates [27]–[29], [46], [51], the former preparation composed predominately of intracellular membranes. Although hydropathy analysis indicates a potential trans-membrane domain at the C terminus of SpARC1 (Fig. S1), our experimental data fail to confirm the prediction. Thus, both Na_2_CO_3_ treatment and hypotonic lysis effect release of SpARC1 from membrane fractions (Fig. 3A). It is clear from our data that SpARC1 enters the endoplasmic reticulum based on its sensitivity to PNGase F. However, because ADP-ribosyl cyclase activity was detectable even when the continuity of the endoplasmic reticulum was maintained (Fig. 8), we considered the possibility that this was a result of translocation of SpARC1 to the cytosolic face of the membrane. As shown in Fig. 3, SpARC1 was resistant to both high salt treatment (which would strip peripherally attached cytosolic proteins) and Proteinase K (which would degrade all exposed proteins). These data provide strong evidence for a luminal location. Despite the evidence that SpARC1 is a soluble endoplasmic reticulum protein, we were unable to detect SpARC1 secretion from *Xenopus laevis* oocytes overexpressing the enzyme (Fig. 4). This finding suggests that SpARC1 does not follow the constitutive secretory pathway. One possibility is that the hydrophobic C terminal domain is involved in retaining the enzyme within the endoplasmic reticulum.

**Figure 9:**
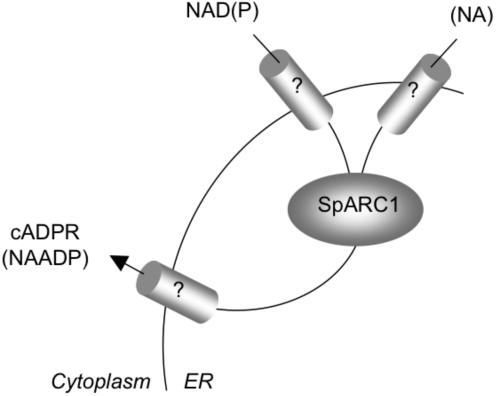
Compartmentalized ADP-ribosyl cyclase signalling. Hypothetical scheme whereby putative transporter molecules allow delivery of substrates from the cytoplasm (*Cyto*.) to SpARC1 located in the endoplasmic reticulum (*ER*) and the subsequent release of the formed products. See text for details.

Because the binding sites for cyclic ADP-ribose and NAADP on their respective calcium channels are more than likely cytosolic, the luminal localization of SpARC1 raises the question of how the substrates enter and products leave the endoplasmic reticulum. As shown in Fig. 8A, SpARC1 clearly can gain access to at least one of its substrates under conditions which preserve luminal continuity. The integrity of the endoplasmic reticulum was confirmed by retention of SpARC1 in pellet fractions upon completion of enzyme assays and the ability of the homogenates to actively accumulate ^45^Ca in a thapsigargin-sensitive manner. It is noteworthy that NAD synthesis occurs largely in the cytoplasm [52], yet many NAD-requiring enzymes are found in organelles. Transporters must therefore exist to effect delivery although molecular details are severely lacking. Of relevance is the recent first molecular cloning of a mitochondrial NAD transporter [53]. Similar transporters for NAD(P) might therefore be present in the endoplasmic reticulum (Fig. 9). Luminal targeting of ADP-ribosyl cyclases could also provide an environment for the accumulation of necessary co-substrates. For example, although *in vitro* experiments have established that ADP-ribosyl cyclases can synthesize NAADP, whether this is the enzymatic route for NAADP synthesis *in vivo* is not clear, because of the high millimolar concentrations of nicotinic acid required to favour the base-exchange reaction (see [54]). Such concentrations are unlikely to be attained extracellularly, or within the cytosol. However, concentrative uptake in to a compartment could in principle achieve the necessary concentrations (Fig. 9). Regarding product release, Deflora and colleagues have shown that both equilibrative and concentrative membrane nucleoside transporters can drive uptake of cyclic ADP-ribose in to cells following its extracellular production [55]. Evidence for a possible NAADP transporter has also been obtained [56], [57]. By analogy, we propose the presence of topologically similar proteins within the endoplasmic reticulum to allow release of products to the cytosol (Fig. 9). Indeed, equilibrative nucleoside transporters have been described in the nuclear and endoplasmic reticulum membranes [58]. Calcium mobilizing messenger levels might therefore be regulated by controlled entry of substrates and/or their release from intracellular compartments. Intriguingly, NAADP-sensitive calcium channels expressed in sea urchin eggs undergo profound inactivation to even sub-threshold (pM) concentrations of NAADP [59], [60], yet measured levels of total cellular NAADP appear to be much higher [46], [47]. This finding is entirely consistent with the compartmentalized synthesis of NAADP.

Calcium signals regulate a whole host of physiological processes [1]. Cyclic ADP-ribose-mediated calcium signals have been implicated in key events such as fertilization of sea urchin eggs [61], [62] and insulin secretion from pancreatic beta cells [63]. Calcium signals mediated by NAADP are also important for fertilization [47], [64] and several calcium-dependent events in neurons such as neurotransmission [65], [66], neuronal growth [67] and differentiation [68]. One prominent feature of all calcium signals is their complexity with respect to both time and space [1]. For example, calcium changes can be generated in one part of the cell and have different physiological consequences to those generated in the other regions [1]. Such complexity is likely key to maintaining specificity given the multitude of stimuli which use calcium to mediate their physiological effects. Our data which suggests that calcium mobilizing messengers may be made within an organelle provides a novel mechanism by which local calcium signals could be generated. This is particularly pertinent given that the endoplasmic reticulum is likely segregated in to functional sub-domains and can undergo marked re-organization [42], [69].

To conclude, molecular identification of sea urchin ADP-ribosyl cyclases reveals a new location for this family of enzymes, which may constitute a highly compartmentalized signaling unit.

## Materials and Methods

### Molecular Cloning of Sea Urchin ADP-Ribosyl Cyclases

Sea urchin ADP-ribosyl cyclase sequences were determined by PCR screening of *Strongylocentrotus purpuratus* cDNA libraries derived from the testes and/or ovary, the latter kindly provided by Professor Gary Wessel, Brown University, USA. We used combinations of vector- and gene-specific primers. Reactions were initially denatured for 2 min at 94°C followed by 35 cycles of denaturation (30 s, 94°C), annealing (30 s at the specified temperature) and extension (3 min, 68°C) using Platinum® *Taq* DNA Polymerase Hi Fidelity (Invitrogen).

A ∼1.4 kbp fragment was amplified from the testes library (annealing temp = 56°C) using T7 primer and a forward gene-specific primer (5′-GCAATGTCAAGTGTCGTG-3′). The latter was based upon a 4849 bp contig (contig 638076) identified by an initial BLAST search of the *Stronglylocentrotus purpuratus* genome sequencing project at the Baylor College of Medicine (http://www.hgsc.bcm.tmc.edu/projects/seaurchin/) with the *Aplysia californica* ADP-ribosyl sequence (accession number: P29241). The amplified product was directly sequenced and found to correspond to the 3′ coding sequence and UTR for a putative ADP-ribosyl cyclase which we termed SpARC1. The 5′ sequence of the library insert was obtained by PCR using T3 primer and the following reverse gene-specific primer located in the 3′ region of the putative open reading frame: 5′-GGCGTATGGCCCACAGTAT-3′ (annealing temp = 56°C). Sequencing of the 900 bp amplified product indicated that the insert was a partial 5′clone. A BLAST search of the obtained sequence identified a second 93959 bp contig (contig 638078) that extended the 5′ sequence of SpARC1 to an in frame stop codon.

A search of EST databases with the putative SpARC1 sequence led to the identification of a larval clone (accession CD306910) corresponding to the 5′ UTR and initial coding sequence of a second homologue termed SpARC2. PCR using a forward primer based in the 5′ UTR (5′-CTGTTTGCATCTAACGCACCT-3′) and T7 primer (annealing temp = 60°C) resulted in the amplification of a 1.8 kbp product from the ovary library. Sequencing of this product indicated that it encompassed the entire open reading frame as evidenced by the presence of two in frame stop codons.

The sequence of a third homologue (SpARC3) was determined based on contig 344575 (5103 bp) identified in the initial BLAST search (see above). A forward gene-specific primer (5′-CGTTCACTCGTATGCCAAAG-3′)+T3 primer resulted in the amplification of an 800 bp product (annealing temp = 55°C), the sequence of which corresponded to the 3′ coding region and UTR. The 5′ region of the insert was determined by sequencing of a 400 bp product amplified at an annealing temperature of 55°C using T7 primer and the following reverse gene-specific primer: 5′-ACCCTTTAGCGAACCCACAT-3′. This insert was found to be a partial 5′ clone. A second reverse gene-specific primer (5′TTGCATGCTTCACGGTACTT-3′) and T7 primer however resulted in the amplification of a 600 bp product (annealing temp = 52°C) from an independent overlapping insert which extended the 5′ sequence to an in frame stop codon. All products were amplified from the testes library.

A BLAST search of the obtained sequences against the genomic nucleotide data indicated 97–98% identity (data not shown). One significant difference was the absence of nucleotides 671–718 of the SpARC2 sequence from the genome assembly. Our nucleotide sequence was however confirmed by two recently published ESTs (accession numbers EC433614 and EC437263). The translational start site for each isoform was predicted by identifying the first in frame ATG codon downstream of the 5′ stop codon that conformed to the Kozak consensus. The predicted coding sequences have been deposited in GenBank under the following accession numbers: AM494973 (SpARC1), AM494974 (SpARC2) and AM494975 (SpARC3).

The full length coding sequence for SpARC1 was amplified at an annealing temperature of 47°C from the ovary library by PCR using the following primer pair: SpARC1 1F (5′-*CACC* AGATCTATGGGCATCTACACCATATTCA-3′)+SpARC1 1R (5′-ACCCAATCGATCTAGGGTAGTAGATATTGTATTCCAACC-3′). Sites introduced for directional TOPO® cloning (italics) and restriction enzymes (underlined) are highlighted. The PCR product was digested with *Bgl*II+*Cla*I and ligated directly in to pCS2+MT (http://sitemaker.umich.edu/dlturner.vectors/home) at the *BamH*I and *Cla*I sites in order to introduce six *myc* tags at the C terminus. The clone was verified by sequencing both strands of DNA.

### General Bioinformatics

Sequence alignments were performed using Clustal-W [70]. Hydropathy plots were generated using the Kyte-Doolittle algorithm [71]. Signal peptides/signal anchors and GPI-anchors were predicted using SignalP version 3.0 [38] and big-PI predictor [39], respectively. Sequences were scanned for N-glycosylation sites using the Prosite database on the ExPASy Proteomics Server.

### *In Vitro* Transcription

Capped mRNA transcripts were prepared using the mMessage mMachine® kit (Ambion) according to the manufacturers instructions. Briefly, plasmids were linearized at the SacII site, purified on QIAprep spin columns (Qiagen) and used as templates for SP6-driven transcription. Reactions were terminated with DNaseI and the transcripts purified using RNeasy mini spin columns (Qiagen).

### *In Vitro* Translation

mRNA was translated using the rabbit reticulocyte lysate system either in the absence or presence of canine pancreatic microsomes (Promega). Typical reactions (25 µl) contained 0.5 µg of mRNA, 17.5 µl of rabbit reticulocyte lysate, 0.5 µl of an amino acid mixture lacking methionine (1 mM), 1 µl of RNasin® RNase inhibitor and 29 µCi of ^35^S -labelled methionine (1000 Ci/mmol; Pro-mix, G.E Healthcare). Where indicated, 2.4 µl of microsomes were added. Translation was performed at 30°C for 90 min and 1 µl aliquots of the reactions separated on NuPAGE® 4–12% Bis-Tris gels (Invitrogen). Autoradiograms were obtained by apposing the dried gels to BioMax MS film (Kodak) for 2–18 h following fixation in 40% methanol and 10% acetic acid (20 min).

### Cell Culture

*Xenopus laevis* eggs were procured and fertilized using standard techniques [40]. Stage VI oocytes were collected from female *Xenopus laevis* and maintained in modified Barth's saline supplemented with 25 µg/ml Gentamicin (Sigma). In some experiments, oocytes were matured *in vitro* with progesterone (1 µg/ml) for 12 hr incubation at 18°C. Human embryonic kidney (HEK) cells were maintained in DMEM supplemented with 10% (v/v) serum and 100 units/ml penicillin and 100 µg/ml streptomycin at 37°C in a humidified atmosphere of 95% air and 5% CO_2_. Media was replaced every 3–4 days.

### Microinjection of *Xenopus Laevis* Embryos and Oocytes

Embryos were injected at the 2-cell stage with 0.8 ng of synthetic mRNA per blastomere and incubated in 5% normal amphibian medium [40], containing 3% Ficoll (Sigma), at 14°C. Embryos were collected at the late blastula stage. Oocytes were defolliculated by incubation with 2 mg/ml Collagenase (Sigma Type 1A) in modified Barth's saline, for 2 hours with agitation. Following overnight culture in fresh media at 14°C, healthy oocytes were injected with 20–30 ng of synthetic capped mRNA. The cells were then maintained for a further 18–20 h in 96 well plates pre-coated with 1 mg/ml BSA (10 oocytes per well in 50 µl media) before harvesting of cells and media. For immunolocalisation experiments, cDNA was microinjected (∼8 ng plasmid) directly into the nucleus of the oocyte.

### Transfection of HEK Cells

Cells were seeded either directly into 6-well plates (for western blotting) or onto 25 mm diameter coverslips (for immunocytochemistry). Surfaces were coated with poly-L-lysine (20 µg/ml). Upon reaching ∼90% confluency, the cells were transiently transfected with 4 µg of pCS2+MT-SpARC1 (see above), pDsRed2-ER (Invitrogen) or a plasmid encoding for the S65T mutant of green fluorescent protein targeted to mitochondria (kindly provided by Prof Rossario Rizzuto, University of Ferrara, Ferrara, Italy) using Lipofectamine^TM^ 2000 transfection reagent (Invitrogen) and according to the manufacturers instructions. Subsequent immunocytochemistry and western blotting experiments were performed 17 h post-transfection.

### Preparation of Cell Homogenates and Deglycosylation

For embryos and oocytes, the cells were homogenised in a medium (20 µl/cell) composed of 50 mM NaCl, 10 mM MgAcetate, 1% v/v Triton X-100, 20 mM Tris (pH 7.2), incubated for 30 min and then clarified by centrifugation at 21,000×g for 1 min. For HEK cells, the adherent cells were washed once with phosphate-buffered saline (10 mM phosphate, 2.7 mM KCl and 137 mM NaCl, pH 7.4) and homogenized (100 µl/10 cm^2^ of confluent culture) in a medium composed of 150 mM NaCl, 1 mM EDTA, 1% v/v Triton X-100, 20 mM HEPES (pH 7.2). All of the above procedures were performed at 4°C using homogenization buffers supplemented with Complete™ EDTA-free protease inhibitors (Roche). For deglycosylation, the homogenates (0.5 embryo equivalents, 25 µg of HEK cell homogenate) were denatured and treated with PNGase F (New England Biolabs) according to the manufacturer's instructions before Western Blot analysis.

### Subcellular Fractionation of *Xenopus laevis* Embryos

Embryos were homogenised in buffer A (20 µl/embryo) composed of 10% w/v sucrose, 150 mM NaCl, 10 mM MgAcetate and 20 mM Tris (pH 7.2) and diluted two-fold either in to the same medium, 200 mM Na_2_CO_3_ (pH 8.5) or a hypertonic medium containing 2 M NaCl and 20 mM Tris (pH 7.2). In some experiments, the embryos were frozen at −20°C, thawed, homogenized in a hypotonic medium composed of 20 mM HEPES (pH 7.2) and then sonicated (3×5 s bursts). All samples were then incubated for 1 h and centrifuged at 100,000×g for an additional 1 h. The supernatant fractions were recovered and the pellets solubilized with a buffer composed of 50 mM NaCl, 10 mM MgAcetate, 1% v/v Triton X-100, 20 mM Tris (pH 7.2) for 30 min. All procedures were performed at 4°C using buffers supplemented with Complete™ protease inhibitors (Roche).

### Proteinase K Treatment of *Xenopus laevis* Embryos

Homogenates from embryos injected with SpARC1 mRNA were prepared in buffer A as described above except that the protease inhibitors were omitted. The samples were then diluted two-fold into the same medium containing a final concentration of 0–50 µg/ml Proteinase K (Sigma) and incubated at 4°C for 1 h. The protease was then inactivated by the sequential addition of 5 mM PMSF (15 min, 4°C) and pre-warmed SDS PAGE sample buffer (95°C, 10 min). In control experiments, the homogenates were first solubilized with 1% v/v Triton X-100 (4°C, 30 min) before protease treatment.

### Western Blotting

Homogenates (0.25–0.5 oocyte/embryo equivalents; 25–50 µg of HEK cell homogenate), or oocyte culture medium (0.5–2 oocyte equivalents) were reduced, separated on NuPAGE® 4–12% Bis-Tris gels (Invitrogen) and transferred to nitrocellulose filters (ProBlott^TM^, Applied Biosystems) according to standard procedures. The filters were then blocked overnight at 4°C with 5% w/v dried skimmed milk in Tris-buffered saline (25 mM Tris, 137 mM NaCl and 2.7 mM KCl, pH 7.4) containing 0.1% v/v Tween® 20 (TBS-T) and sequentially incubated with anti-c-*myc* monoclonal antibody (clone 9e10 from Autogen Bioclear; 1/1000 dilution) and anti-mouse IgG conjugated to horse-radish peroxidase (1/1000 dilution) in TBS-T supplemented with 2.5% w/v dried skimmed milk for 1h at room temperature. After each step, the filters were washed with TBS-T (3×30 min). The resulting blots were developed using the ECL^TM^ Advanced system (Amersham) according to the manufacturer's instructions.

### Immunocytochemistry

For oocytes, the cells were fixed by addition of ice cold 1% formaldehyde in methanol (1 ml/5–10 oocytes) and incubated at −20°C for 2 hrs. The samples were then warmed to room temperature and incubated with phosphate-buffered saline supplemented with 0.1% v/v Triton X-100 (PBS-T) for 1 h at room temperature following sequential washes (5 min) in 75% methanol/25% PBS-T, 50% methanol/50% PBS-T and 25% methanol 75% PBS-T. The oocytes were blocked with phosphate-buffered saline supplemented with 4% w/v bovine serum albumin for 2–4 h at room temperature and incubated at 4°C overnight with anti-c-myc monoclonal antibody (myc-tag 9B11 Cell Signaling Technology 1∶1000 dilution) in phosphate-buffered saline supplemented with 2% w/v bovine serum albumin. Following a 1 h wash with phosphate-buffered saline (with several changes of solution), the oocytes were incubated with anti-mouse IgG conjugated to FITC or rhodamine red (1/200 dilution) for 2 h at room temperature. The samples were again washed with phosphate-buffered saline for 1 h and sealed onto glass coverslips using mounting media (Ted Pella Inc.).

For HEK cells, the cells were fixed in paraformaldehyde (4% w/v) at 25°C for 10 min and following three washes in phosphate-buffered saline, permeabilised with Triton X-100 (0.1% v/v, 10 minutes, 25°C). Cells were washed again (three times with phosphate-buffered saline) and then incubated in blocking buffer consisting of phosphate-buffered saline supplemented with fetal bovine serum (5% v/v) and bovine serum albumin (1% w/v) for 1 h at 25°C. Incubation of primary and secondary antibodies (1/100 dilution) was performed sequentially in blocking buffer at 37°C for 1 h. Unbound antibody was removed after each incubation by washing the coverslips three times in PBS-T. The primary antibody used was an anti-c-*myc* monoclonal antibody (clone 9e10 from Autogen Bioclear). Antibody binding was visualised using a secondary antibody (polyclonal, Cy5^TM^-conjugated anti-mouse IgG from goat; Zymed). Coverslips were mounted onto slides with 1,4-diazabicyclo[2.2.2]octane (DABCO) and sealed immediately.

### Confocal Microscopy

Images of oocytes and eggs were captured using a BioRad MRC1024 confocal scanner attached to an Olympus AX70 microscope equipped with a ×60 oil-immersion objective. Images of HEK cells were captured using a Zeiss LSM 510 confocal microscope (Welwyn Garden city, Herts, UK) using conventional filter-based fluorescence optics. The respective excitation and emission wavelengths were 488 nm and 505–550 nm for mitochondrial GFP, 543 nm and 560–615 nm for DsRed2-ER and 633 nm and >650 nm for Cy5.

### Enzyme activity measurements

*Xenopus laevis* embryos were homogenized (4.5 ul/embryo) in media containing Complete™ EDTA-free protease inhibitors (Roche) and either 20 mM HEPES (pH 7.2) followed by sonication (3×5 s bursts) or buffer A. The homogenates prepared in buffer A were used immediately whereas the hypotonic lysates were used either immediately or after freezing at −20°C.

For base-exchange activity measurements, hypotonic lysates (typically 50 µl) were diluted 5-fold in to a medium composed of 1 mM NADP and 50 mM nicotinic acid (pH 4). For measurement of cyclase activity both hypotonic lysates or homogenates freshly-prepared in the presence of sucrose (buffer A) were used. In these experiments, the homogenates were diluted 5-fold in to the same medium used for their preparation to which 1 mM NGD had been added. All reactions were allowed to proceed for 3–21 h at room temperature. At the appropriate time, samples were diluted 10-fold with H_2_O and particulate material removed by centrifugation (12,000×g, 1 min). The supernatants were analyzed for nucleotide content by anion exchange HPLC using a 3×150 mm column packed with AGMP1 (Biorad) as described [72] with minor modification. The column was washed with H_2_O for 2 min at a flow rate of 1 ml/min upon injection and bound nucleotides eluted using a gradient of trifluoroacetic acid that increased linearly to 2% from 2–8 min, to 4% at 14 min, to 8% at 20 min, to 16% at 26 min, to 32% at 32 min, to 64% at 38 min and to 100% (150 mM trifluoroacetic acid) at 42 min. Absorbance was monitored at 254 nm and area measurements of the resolved peaks determined using Breeze™ Software (Waters). Elution was compared to authentic NAADP and cyclic GDP ribose. The latter was synthesized by incubation of NGD (1 mM) with 2–100 ng/ml *Aplysia* ADP-ribosyl cyclase (Sigma) for 1–21 h at room temperature in a buffer containing 20 mM HEPES, pH 7.2.

### ^45^Ca flux

*Xenopus* embryo homogenates were prepared in sucrose-containing buffer A as described above. The homogenates were then sequentially diluted (2-fold every 30 minutes, room temperature) to a final concentration of 22.75 µl/embryo. The dilution buffer used was buffer A supplemented with 1.5 µM ^45^CaCl_2_ (specific activity = 1Ci/mmol; G.E. Healthcare), 3 µg/ml oligomycin+3 µg/ml antimycin (to inhibit mitochondrial uptake) and an ATP regeneration system composed of 1 mM MgATP, 10 mM phosphocreatine and 10 U/ml creatine phosphokinase. Incubations were performed either with or without thapsigargin (1 µM). After 3 h the samples were quenched by rapid filtration through Whatman GF-B filters under vacuum followed by three washes with an ice-cold buffer composed of 300 mM sucrose and 3 mM EGTA (pH 7.2). Radioactivity associated with the filters was determined by scintillation counting. Non-specific binding of the radiolabel was determined in the presence of 10 µM ionomycin. In parallel experiments, we assayed ^45^Ca uptake in to sea urchin egg homogenates (3.125% v/v final) that were prepared as described previously [73]. Experimental conditions were the same as those for the *Xenopus* embryo homogenates except that an intracellular-like medium composed of 250 mM potassium gluconate, 250 mM N-methyl-D-glucamine, 1 mM MgCl_2_ and 20 mM HEPES (pH 7.2, with acetic acid) was used.

## Supporting Information

Figure S1 Hydropathy analysis. Kyte-Doolittle plots of the amino acid sequences of sea urchin ADP-ribosyl cyclases. A window size of 19 was used for the analysis.
(4.54 MB TIF)

Table S1 Amino acid sequence homology between SpARCs and other members of the ADP-ribosyl cyclase family. Tabulated values were calculated from pair-wise alignments. Percentage identities of the sequences are listed below the diagonal and percentage similarities above. Abbreviations: Hs, Homo sapiens; Rn, Rattus norwegicus; Mm, Mus musculus, Oc, Oryctolagus cuniculus; Cf, Canis familiaris; Bt, Bos taurus; Ac; Aplysia californica; Ak, Aplysia kurodai; Sm. Schistosoma mansoni.
(0.06 MB DOC)

Table S2 Predicted post translation modifications for sea urchin ADP-ribosyl cyclases. The amino acid sequence for each ADP-ribosyl cyclase was inspected for the indicated post-translational modification using the algorithms described in the Methods. Positive predictions are underlined.
(0.01 MB RTF)

## References

[1] Berridge MJ, Lipp P, Bootman MD (2000). The versatility and universality of calcium signalling.. Nat Rev Mol Cell Biol.

[2] Clapper DL, Walseth TF, Dargie PJ, Lee HC (1987). Pyridine nucleotide metabolites stimulate calcium release from sea urchin egg microsomes desensitized to inositol trisphosphate.. J Biol Chem.

[3] Lee HC (2004). Multiplicity of Ca2+ messengers and Ca2+ stores: a perspective from cyclic ADP-ribose and NAADP.. Curr Mol Med.

[4] Patel S, Churchill GC, Galione A (2001). Coordination of Ca^2+^ signalling by NAADP.. Trends Biochem Sci.

[5] Galione A, Lee HC, Busa WB (1991). Ca(2+)-induced Ca2+ release in sea urchin egg homogenates: modulation by cyclic ADP-ribose.. Science.

[6] Berridge G, Dickinson G, Parrington J, Galione A, Patel S (2002). Solubilization of receptors for the novel Ca^2+^-mobilizing messenger, nicotinic acid adenine dinucleotide phosphate.. J Biol Chem.

[7] Churchill GC, Okada Y, Thomas JM, Genazzani AA, Patel S (2002). NAADP mobilizes Ca^2+^ from reserve granules, lysosome-related organelles, in sea urchin eggs.. Cell.

[8] Galione A, Petersen OH (2005). The NAADP Receptor: New Receptors or New Regulation?. Mol Interv.

[9] Lee HC (2000). Enzymatic functions and structures of CD38 and homologs.. Chem Immunol.

[10] Glick DL, Hellmich MR, Beushausen S, Tempst P, Bayley H (1991). Primary structure of a molluscan egg-specific NADase, a second-messenger enzyme.. Cell Regul.

[11] Howard M, Grimaldi JC, Bazan JF, Lund FE, Santos-Argumedo L (1993). Formation and hydrolysis of cyclic ADP-Ribose catalysed by lymphocyte antigen CD38.. Science.

[12] Hirata Y, Kimura N, Sato K, Oshugi Y, Takasawa S (1994). ADP-ribosyl cyclase activity of a novel bone marrow stromal cell surface molecule, BST-1.. FEBS Lett.

[13] Goodrich SP, Muller-Steffner H, Osman A, Moutin MJ, Kusser K (2005). Production of Calcium-Mobilizing Metabolites by a Novel Member of the ADP-Ribosyl Cyclase Family Expressed in Schistosoma mansoni.. Biochemistry.

[14] Lee HC (2001). Physiological functions of cyclic ADP-ribose and NAADP as calcium messengers.. Annu Rev Pharmacol Toxicol.

[15] De Flora A, Zocchi E, Guida L, Franco L, Bruzzone S (2004). Autocrine and paracrine calcium signaling by the CD38/NAD+/cyclic ADP-ribose system.. Ann N Y Acad Sci.

[16] Zocchi E, Franco L, Guida L, Piccini D, Tacchetti C (1996). NAD+-dependent internalization of the transmembrane glycoprotein CD38 in human Namalwa B cells.. FEBS Lett.

[17] Adebanjo OA, Anandatheerthavarada HK, Koval AP, Moonga BS, Biswas G (1999). A new function for CD38/ADP-ribosyl cyclase in nuclear Ca2+ homeostasis.. Nat Cell Biol.

[18] Kato I, Yamamoto Y, Fujimura M, Noguchi N, Takasawa S (1999). CD38 disruption impairs glucose-induced increases in cyclic ADP-ribose, [Ca^2+^]*_i_* and insulin secretion.. J Biol Chem.

[19] Fukushi Y, Kato I, Takasawa S, Sasaki T, Ong BH (2001). Identification of cyclic ADP-ribose-dependent mechanisms in pancreatic muscarinic Ca(2+) signaling using CD38 knockout mice.. J Biol Chem.

[20] Partida-Sanchez S, Cockayne DA, Monard S, Jacobson EL, Oppenheimer N (2001). Cyclic ADP-ribose production by CD38 regulates intracellular calcium release, extracellular calcium influx and chemotaxis in neutrophils and is required for bacterial clearance in vivo.. Nat Med.

[21] Ceni C, Muller-Steffner H, Lund F, Pochon N, Schweitzer A (2003). Evidence for an intracellular ADP-ribosyl cyclase/NAD+-glycohydrolase in brain from CD38-deficient mice.. J Biol Chem.

[22] Meszaros LG, Wrenn RW, Varadi G (1997). Sarcoplasmic reticulum-associated and protein kinase C-regulated ADP-ribosyl cyclase in cardiac muscle.. Biochem Biophys Res Commun.

[23] Wilson HL, Dipp M, Thomas JM, Lad C, Galione A (2001). ADP-ribosyl Cyclase and Cyclic ADP-ribose Hydrolase Act as a Redox Sensor.. J Biol Chem.

[24] Hohenegger M, Suko J, Gscheidlinger R, Drobny H, Zidar A (2002). Nicotinic acid-adenine dinucleotide phosphate activates the skeletal muscle ryanodine receptor.. Biochem J.

[25] Sternfeld L, Krause E, Guse AH, Schulz I (2003). Hormonal control of ADP-ribosyl cyclase activity in pancreatic acinar cells from rats.. J Biol Chem.

[26] Xie GH, Rah SY, Kim SJ, Nam TS, Ha KC (2005). ADP-ribosyl cyclase couples to cyclic AMP signaling in the cardiomyocytes.. Biochem Biophys Res Commun.

[27] Galione A, White A, Willmott N, Turner M, Potter BVL (1993). cGMP mobilizes intracellular Ca^2+^ in sea urchin eggs by stimulating cyclic ADP-ribose synthesis.. Nature.

[28] Graeff RM, Franco L, De Flora A, Lee HC (1998). Cyclic GMP-dependent and -independent effects on the synthesis of the calcium messengers cyclic ADP-ribose and nicotinic acid adenine dinucleotide phosphate.. J Biol Chem.

[29] Wilson HL, Galione A (1998). Differential regulation of nicotinic acid-adenine dinucleotide phosphate and cADP-ribose production by cAMP and cGMP.. Biochem J.

[30] Guse AH, da Silva CP, Berg I, Skapeno AL, Weber K (1999). Regulation of calcium signalling in T lymphocytes by the second messenger cyclic ADP-ribose.. Nature.

[31] Bruzzone S, De Flora A, Usai C, Graeff R, Lee HC (2003). Cyclic ADP-ribose is a second messenger in the lipopolysaccharide-stimulated proliferation of human peripheral blood mononuclear cells.. Biochem J.

[32] Zhang F, Zhang G, Zhang AY, Koeberl MJ, Wallander E (2006). Production of NAADP and its role in Ca2+ mobilization associated with lysosomes in coronary arterial myocytes.. Am J Physiol Heart Circ Physiol.

[33] Billington RA, Harper C, Bellomo EA, Publicover S, Barratt CL (2006). Characterization of cyclic adenine dinucleotide phosphate ribose levels in human spermatozoa.. Fertil Steril.

[34] Matsumura N, Tanuma S (1998). Involvement of cytosolic NAD+ glycohydrolase in cyclic ADP-ribose metabolism.. Biochem Biophys Res Commun.

[35] Lee HC (1996). Cyclic ADP-ribose and calcium signaling in eggs.. Biol Signals.

[36] Galione A, Patel S, Churchill GC (2000). NAADP-induced calcium release in sea urchin eggs.. Biol Cell.

[37] Munshi C, Thiel DJ, Mathews II, Aarhus R, Walseth TF (1999). Characterization of the active site of ADP-ribosyl cyclase.. J Biol Chem.

[38] Bendtsen JD, Nielsen H, von HG, Brunak S (2004). Improved prediction of signal peptides: SignalP 3.0.. J Mol Biol.

[39] Eisenhaber B, Bork P, Eisenhaber F (1999). Prediction of potential GPI-modification sites in proprotein sequences.. J Mol Biol.

[40] Sive HL, Grainger RM, Harland RM (2000). Early development of *Xenopus laevis*: a laboratory manual.

[41] Subramanian VS, Marchant JS, Parker I, Said HM (2001). Intracellular trafficking/membrane targeting of human reduced folate carrier expressed in Xenopus oocytes.. Am J Physiol Gastrointest Liver Physiol.

[42] Boulware MJ, Marchant JS (2005). IP3 receptor activity is differentially regulated in endoplasmic reticulum subdomains during oocyte maturation.. Curr Biol.

[43] Graeff RM, Walseth TF, Fryxell K, Branton WD, Lee HC (1994). Enzymatic synthesis and characterizations of cyclic GDP-ribose. A procedure for distinguishing enzymes with ADP-ribosyl cyclase activity.. J Biol Chem.

[44] Lee HC, Walseth TF, Bratt GT, Hayers R, Clapper DL (1989). Structural determination of a cyclic metabolite of NAD^+^ with intracellular Ca^2+^-mobilizing activity.. J Biol Chem.

[45] Lee HC, Aarhus R (1995). A derivative of NADP mobilizes calcium stores insensitive to inositol trisphosphate and cyclic ADP-ribose.. J Biol Chem.

[46] Billington RA, Ho A, Genazzani AA (2002). Nicotinic acid adenine dinucleotide phosphate (NAADP) is present at micromolar concentrations in sea urchin spermatozoa.. J Physiol.

[47] Churchill GC, ÒNeil JS, Masgrau R, Patel S, Thomas JM (2003). Sperm deliver a new messenger:NAADP.. Curr Biol.

[48] Kuhn I, Kellenberger E, Rognan D, Lund FE, Muller-Steffner H (2006). Redesign of Schistosoma mansoni NAD+ catabolizing enzyme: active site H103W mutation restores ADP-ribosyl cyclase activity.. Biochemistry.

[49] Moreno-Garcia ME, Sumoza-Toledo A, Lund FE, Santos-Argumedo L (2005). Localization of CD38 in murine B lymphocytes to plasma but not intracellular membranes.. Mol Immunol.

[50] Yamada M, Mizuguchi M, Otsuka N, Ikeda K, Takahashi H (1997). Ultrastructural localization of CD38 immunoreactivity in rat brain.. Brain Res.

[51] Chini EN, Thompson MA, Chini CC, Dousa TP (1997). Cyclic ADP-ribose signaling in sea urchin gametes: metabolism in spermatozoa.. Am J Physiol.

[52] Berger F, Ramirez-Hernandez MH, Ziegler M (2004). The new life of a centenarian: signalling functions of NAD(P).. Trends Biochem Sci.

[53] Todisco S, Agrimi G, Castegna A, Palmieri F (2006). Identification of the mitochondrial NAD+ transporter in Saccharomyces cerevisiae.. J Biol Chem.

[54] Soares S, Thompson M, White T, Isbell A, Yamasaki M (2006). NAADP as a second messenger: Neither CD38 nor the base-exchange reaction are necessary for the in vivo generation of the NAADP in myometrial cells.. Am J Physiol Cell Physiol.

[55] Guida L, Bruzzone S, Sturla L, Franco L, Zocchi E (2002). Equilibrative and concentrative nucleoside transporters mediate influx of extracellular cyclic ADP-ribose into 3T3 murine fibroblasts.. J Biol Chem.

[56] Heidemann AC, Schipke CG, Kettenmann H (2005). Extracellular application of nicotinic acid adenine dinucleotide phosphate induces Ca2+ signaling in astrocytes in situ.. J Biol Chem.

[57] Billington RA, Bellomo EA, Floriddia EM, Erriquez J, Distasi C (2006). A transport mechanism for NAADP in a rat basophilic cell line.. FASEB J.

[58] Mani RS, Hammond JR, Marjan JM, Graham KA, Young JD (1998). Demonstration of equilibrative nucleoside transporters (hENT1 and hENT2) in nuclear envelopes of cultured human choriocarcinoma (BeWo) cells by functional reconstitution in proteoliposomes.. J Biol Chem.

[59] Aarhus R, Dickey DM, Graeff R, Gee KR, Walseth TF (1996). Activation and inactivation of Ca^2+^ release by NAADP^+^.. J Biol Chem.

[60] Genazzani AA, Empson RM, Galione A (1996). Unique inactivation properties of NAADP-sensitive Ca^2+^ release.. J Biol Chem.

[61] Galione A, McDougall A, Busa WB, Willmott N, Gillot I (1993). Redundant mechanisms of calcium-induced calcium release underlying calcium waves during fertilization of sea urchin eggs.. Science.

[62] Lee HC, Aarhus R, Walseth TF (1993). Calcium mobilization by dual receptors during fertilization of sea urchin eggs.. Science.

[63] Takasawa S, Nata K, Yonekura H, Okamoto H (1993). Cyclic ADP-ribose in insulin secretion from pancreatic beta cells.. Science.

[64] Lim D, Kyozuka K, Gragnaniello G, Carafoli E, Santella L (2001). NAADP^+^ initiates the Ca^2+^ response during fertilization of starfish oocytes.. FASEB J.

[65] Chameau P, Van de Vrede Y, Fossier P, Baux G (2001). Ryanodine-, IP_3_- and NAADP-dependent calcium stores control acetylcholine release.. Pflƒg Archiv.

[66] Brailoiu E, Miyamoto MD, Dun NJ (2001). Nicotinic acid adenine dinucleotide phosphate enhances quantal neurosecretion at the frog neuromuscular junction: possible action on synaptic vesicles in the releasable pool.. Mol Pharmacol.

[67] Brailoiu E, Hoard JL, Filipeanu CM, Brailoiu GC, Dun SL (2005). NAADP potentiates neurite outgrowth.. J Biol Chem.

[68] Brailoiu E, Churamani D, Pandey V, Brailoiu GC, Tuluc F (2006). Messenger-specific role for NAADP in neuronal differentiation.. J Biol Chem.

[69] Terasaki M, Jaffe LA (1991). Organization of the sea urchin egg endoplasmic reticulum and its reorganization at fertilization.. J Cell Biol.

[70] Chenna R, Sugawara H, Koike T, Lopez R, Gibson TJ (2003). Multiple sequence alignment with the Clustal series of programs.. Nucleic Acids Res.

[71] Kyte J, Doolittle RF (1982). A simple method for displaying the hydropathic character of a protein.. J Mol Biol.

[72] Berridge G, Cramer R, Galione A, Patel S (2002). Metabolism of the novel Ca^2+^-mobilizing messenger nicotinic acid-adenine dinucleotide phosphate via a 2′-specific Ca^2+^-dependent phosphatase.. Biochem J.

[73] Dickinson GD, Patel S (2003). Modulation of NAADP receptors by K^+^ ions: Evidence for multiple NAADP receptor conformations.. Biochem J.

