# Supplementary figures and images for: Molecular Characterization of a Novel Intracellular ADP-Ribosyl Cyclase
Source: PLoS One. 2007 Aug 29;2(8):e797. doi: 10.1371/journal.pone.0000797 (PMC1949048; doi:10.1371/journal.pone.0000797)

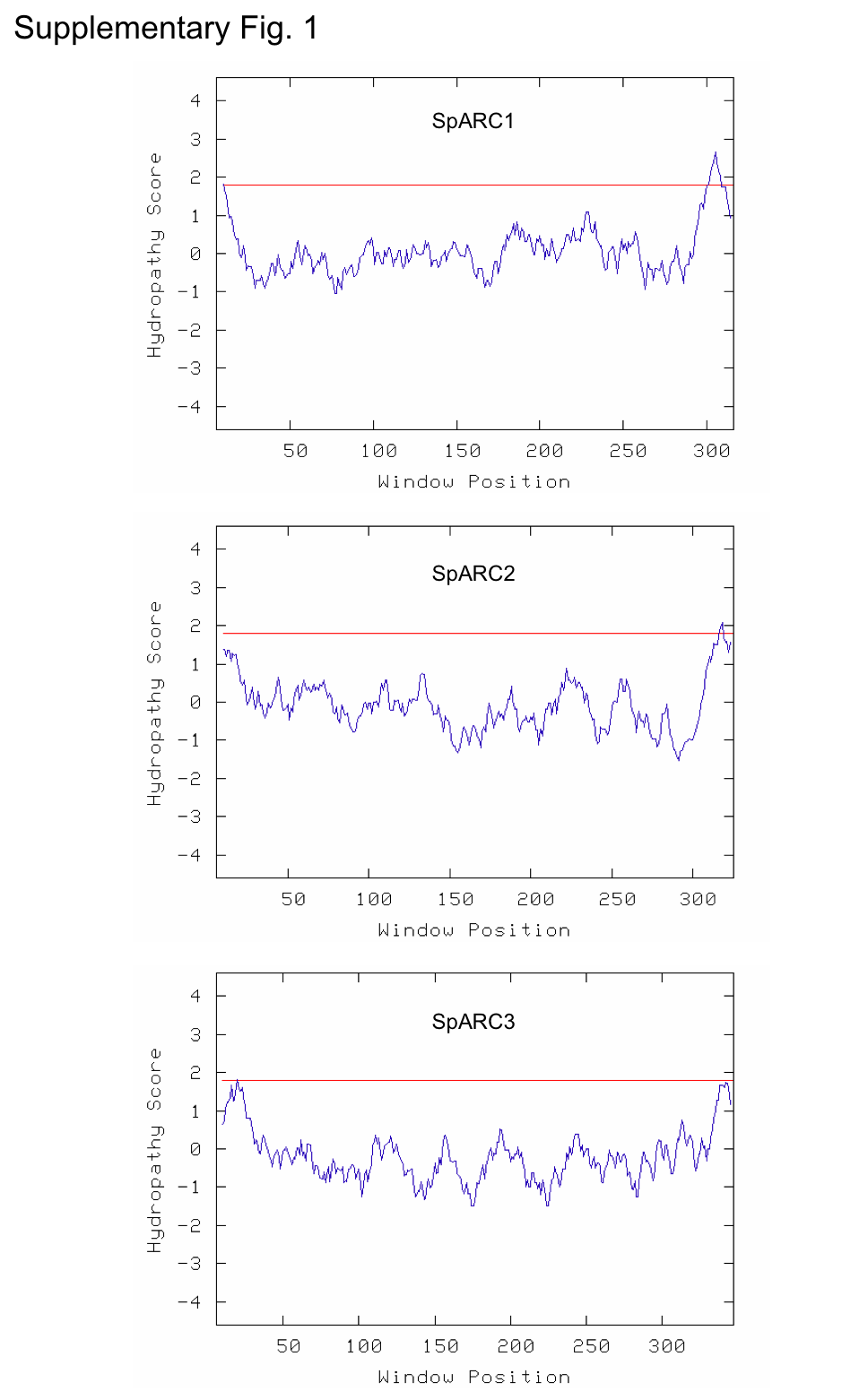

Supplement: Figure S1 — Hydropathy analysis. Kyte-Doolittle plots of the amino acid sequences of sea urchin ADP-ribosyl cyclases. A window size of 19 was used for the analysis. (4.54 MB TIF) [file pone.0000797.s001.tif]
