# Supplementary material for: Molecular Characterization of a Novel Intracellular ADP-Ribosyl Cyclase
Source: PLoS One. 2007 Aug 29;2(8):e797. doi: 10.1371/journal.pone.0000797 (PMC1949048; doi:10.1371/journal.pone.0000797)
Supplement: Table S1 — Amino acid sequence homology between SpARCs and other members of the ADP-ribosyl cyclase family. Tabulated values were calculated from pair-wise alignments. Percentage identities of the sequences are listed below the diagonal and percentage similarities above. Abbreviations: Hs, Homo sapiens; Rn, Rattus norwegicus; Mm, Mus musculus, Oc, Oryctolagus cuniculus; Cf, Canis familiaris; Bt, Bos taurus; Ac; Aplysia californica; Ak, Aplysia kurodai; Sm. Schistosoma mansoni. (0.06 MB DOC) [file pone.0000797.s002.doc]

| **SpARC1** | **SpARC2** | **SpARC3** | **AcARC** | **AkARC** | **HsCD38** | **RnCD38** | **MmCD38** | **OcCD38** | **CfCD38** | **BtCD38** | **HsCD157** | **RnCD157** | **MmCD157** | **SmNACE** |  |
| --- | --- | --- | --- | --- | --- | --- | --- | --- | --- | --- | --- | --- | --- | --- | --- |
| **100** | 66 | 34 | 40 | 39 | 34 | 37 | 35 | 37 | 37 | 33 | 41 | 39 | 38 | 37 | **SpARC1** |
| *51* | **100** | 36 | 37 | 38 | 34 | 36 | 36 | 37 | 35 | 33 | 38 | 35 | 38 | 35 | **SpARC2** |
| *21* | *18* | **100** | 38 | 40 | 35 | 35 | 36 | 36 | 37 | 31 | 39 | 37 | 37 | 32 | **SpARC3** |
| *25* | *23* | *24* | **100** | 92 | 40 | 41 | 41 | 40 | 40 | 39 | 42 | 41 | 43 | 35 | **AcARC** |
| *26* | *24* | *24* | *86* | **100** | 40 | 41 | 40 | 40 | 40 | 39 | 43 | 43 | 43 | 35 | **AkARC** |
| *18* | *20* | *20* | *26* | *26* | **100** | 71 | 69 | 68 | 68 | 53 | 44 | 44 | 43 | 34 | **HsCD38** |
| *20* | *20* | *21* | *29* | *28* | *57* | **100** | 91 | 71 | 71 | 53 | 45 | 42 | 42 | 35 | **RnCD38** |
| *20* | *21* | *21* | *29* | *28* | *57* | *87* | **100** | 70 | 69 | 53 | 47 | 44 | 45 | 37 | **MmCD38** |
| *19* | *19* | *20* | *27* | *26* | *50* | *58* | *58* | **100** | 67 | 56 | 45 | 41 | 41 | 35 | **OcCD38** |
| *20* | *21* | *21* | *27* | *26* | *55* | *58* | *57* | *50* | **100** | 57 | 46 | 43 | 43 | 36 | **CfCD38** |
| *21* | *20* | *19* | *24* | *24* | *39* | *39* | *39* | *39* | *43* | **100** | 41 | 39 | 39 | 32 | **BtCD38** |
| *24* | *23* | *23* | *26* | *25* | *31* | *31* | *32* | *28* | *33* | *25* | **100** | 81 | 80 | 35 | **HsCD157** |
| *22* | *21* | *21* | *24* | *24* | *28* | *27* | *28* | *26* | *29* | *25* | *72* | **100** | 90 | 36 | **RnCD157** |
| *22* | *23* | *21* | *26* | *25* | *28* | *29* | *30* | *27* | *29* | *25* | *71* | *87* | **100** | 37 | **MmCD157** |
| *20* | *16* | *18* | *20* | *20* | *20* | *21* | *21* | *22* | *21* | *20* | *19* | *18* | *20* | **100** | **SmNACE** |

Supplementary Table 1. **Amino acid sequence homology between SpARCs and other members of the ADP-ribosyl cyclase family**. Tabulated values were calculated from pair-wise alignments**.** Percentage identities of the sequences are listed below the diagonal and percentage similarities above. Abbreviations*:* Hs, *Homo sapiens*; Rn*, Rattus norwegicus*; Mm*, Mus musculus*, Oc, *Oryctolagus cuniculus*; Cf, *Canis familiaris*; Bt, *Bos taurus*; Ac; *Aplysia californica*; Ak, *Aplysia kurodai*; Sm. *Schistosoma mansoni.*
