# Supplementary material for: Molecular Characterization of a Novel Intracellular ADP-Ribosyl Cyclase
Source: PLoS One. 2007 Aug 29;2(8):e797. doi: 10.1371/journal.pone.0000797 (PMC1949048; doi:10.1371/journal.pone.0000797)
Supplement: Table S2 — Predicted post translation modifications for sea urchin ADP-ribosyl cyclases. The amino acid sequence for each ADP-ribosyl cyclase was inspected for the indicated post-translational modification using the algorithms described in the Methods. Positive predictions are underlined. (0.01 MB RTF) [file pone.0000797.s003.rtf]

	
SpARC1
	
SpARC2
	
SpARC3	

Probability of a signal peptide 

Probability of a signal anchor 

GPI anchor score

Number of consensus N-glycosylation sites 
	
0.862


0.012


-24.81

7	
0.994


0.005


-2.65

5	
0.082


0.896


3

8	

Supplementary Table 2. Predicted post translation modifications for sea urchin ADP-ribosyl cyclases. The amino acid sequence for each ADP-ribosyl cyclase was inspected for the indicated post-translational modification using the algorithms described in the Methods. Positive predictions are underlined
